# Breast cancer receptor status and stage at diagnosis in over 1,200 consecutive public hospital patients in Soweto, South Africa: a case series

**DOI:** 10.1186/bcr3478

**Published:** 2013-09-17

**Authors:** Valerie A McCormack, Maureen Joffe, Eunice van den Berg, Nadine Broeze, Isabel dos Santos Silva, Isabelle Romieu, Judith S Jacobson, Alfred I Neugut, Joachim Schüz, Herbert Cubasch

**Affiliations:** 1 International Agency for Research on Cancer, 150 Cours Albert Thomas, Lyon 69008, France; 2 Chris Hani Baragwanath Academic Hospital Breast Clinic, Old Potch Road, 2013, Soweto, South Africa; 3 National Health Laboratory Services (NHLS), Johannesburg, South Africa; 4 Department of Non-Communicable Disease Epidemiology, London School of Hygiene and Tropical Medicine, Keppel St, London WC1E 7HT, UK; 5 Departments of Medicine and Epidemiology and Herbert Irving Comprehensive Cancer Center, Columbia University, 722 West 168th Street, New York, NY 10032, USA

## Abstract

**Introduction:**

Estimates of the proportion of estrogen receptor negative (ERN) and triple-negative (TRN) breast cancer from sub-Saharan Africa are variable and include high values. Large studies of receptor status conducted on non-archival tissue are lacking from this region.

**Methods:**

We identified 1218 consecutive women (91% black) diagnosed with invasive breast cancer from 2006–2012 at a public hospital in Soweto, South Africa. Immunohistochemistry based ER, progesterone receptor (PR) and human epidermal factor 2 (HER2) receptors were assessed at diagnosis on pre-treatment biopsy specimens. Mutually adjusted associations of receptor status with stage, age, and race were examined using risk ratios (RRs). ER status was compared with age-stratified US Surveillance Epidemiology and End Results program (SEER) data.

**Results:**

35% (95% confidence interval (CI): 32–38) of tumors were ERN, 47% (45–52) PRN, 26% (23–29) HER2P and 21% (18–23) TRN. Later stage tumors were more likely to be ERN and PRN (RRs 1.9 (1.1-2.9) and 2.0 (1.3-3.1) for stage III vs. I) but were not strongly associated with HER2 status. Age was not strongly associated with ER or PR status, but older women were less likely to have HER2P tumors (RR, 0.95 (0.92-0.99) per 5 years). During the study, stage III + IV tumors decreased from 66% to 46%. In black women the percentage of ERN (37% (34–40)) and PRN tumors (48% (45–52)) was higher than in non-black patients (22% (14–31) and 34% (25–44), respectively, *P* = 0.004 and *P* = 0.02), which remained after age and stage adjustment. Age-specific ERN proportions in black South African women were similar to those of US black women, especially for women diagnosed over age 50.

**Conclusion:**

Although a greater proportion of black than non-black South African women had ER-negative or TRN breast cancer, in all racial groups in this study breast cancer was predominantly ER-positive and was being diagnosed at earlier stages over time. These observations provide initial indications that late-stage aggressive breast cancers may not be an inherent feature of the breast cancer burden across Africa.

## Introduction

Breast cancer receptor status, most commonly defined by estrogen-receptor (ER), progesterone-receptor (PR), and human epidermal growth factor receptor 2 (HER2) status in the clinical setting, has major implications for breast cancer prevention strategies and patient management [1,2]. Studies of these markers in African women with breast cancer in sub-Saharan Africa (SSA) have had extremely variable findings; reported percentages of estrogen receptor negative (ERN) tumors range from 30% to 40% [3-5] to >70% [6-9]; in comparison, corresponding percentages in the United States are 35% in breast cancer patients aged 40 and decline to 15% to 20% by age 70, and are slightly higher in black than in white American women [10]. In SSA, for example, in 75 Ghanaian breast cancer patients, 76% were (ERN) based on receptor testing carried out on formalin-fixed paraffin-embedded specimens obtained in Ghana and transported to the United States for receptor assessment [9]. Similarly, in 500 tumor blocks from Nigeria and Senegal, half were triple negative [7]. At the other end of the spectrum, 27% of tumors were ERN among 192 Nigerian breast cancer patients in a setting in which immunohistochemistry (IHC) was routinely conducted prospectively at diagnosis [3]. The latter study is consistent with recent related results from breast cancers diagnosed in the United States in African-born women, of which 30% of tumors with known receptor status were ERN [11].

A well-known pitfall of ER testing is that results are highly sensitive to biopsy-tissue fixation and processing procedures. These factors have led to false negatives worldwide, as highlighted in the Consensus Recommendations on Estrogen Receptor Testing in Breast Cancer by Immunohistochemistry [12]. Ideally, receptor status is determined on biopsy specimens obtained before preoperative neoadjuvant chemotherapy, and IHC is conducted within a short time-frame to avoid antigen degradation [13]. Receptor-status data from some previous SSA studies may have been vulnerable to such biases because of a lack of routine receptor testing conducted at diagnosis. Additionally, if receptor status is determined from mastectomy tissue taken after neoadjuvant chemotherapy, the tumor phenotype may have evolved from its original status. Conversely, overall ERN proportions may be relatively high because of the relatively young population age-structure; ERN disease is more common in younger cases.

Knowledge of the receptor-status distribution among breast cancer patients in SSA is needed, given the uncertainty present and to improve our understanding of tumor biology, prevention targets, and prognosis. To begin to meet this need, we analyzed unique data from a large (>1,200) consecutive breast cancer case series from a public setting in South Africa where receptor status, including HER2, was routinely measured at diagnosis on prechemotherapy biopsy specimens. We examined breast cancer receptor status in relation to demographic and clinical characteristics and compared age-stratified ER status in black South African women with that in US white and black women. A formal assessment of missing receptor status, and racial comparisons between black, white, colored, and Asian South African women were also performed.

## Methods

### Setting

The study was set at the Chris Hani Baragwanath Academic Hospital (CHBAH), a large tertiary referral public hospital (about 3,200 beds) in Soweto, South Africa. South Africa’s public health sector serves 80% of the population. It has a hierarchical referral system; most patients seen at tertiary care facilities are referred from primary health care clinics and secondary referral hospitals [14]. CHBAH serves a population concentrated within approximately 50 km of the hospital, situated south of Johannesburg, Gauteng Province. A specialized breast clinic was initiated at CHBAH in 2000, for which a small diagnostic/treatment fee (R40 ≈ $5) is waived if patients have no means to pay. Most breast cancer patients are symptomatic on arrival, as the majority of women have no access to any form of early-detection efforts such as by screening mammography or routine clinical breast examination; mammographic screening is available only to patients with good private health insurance coverage, and few such patients come to CHBAH for diagnosis. Opportunistic screening on mammography trucks occasionally occurs in Soweto (for example, by PinkDrive since late 2012), but these services were not operational during the period of case ascertainment.

Diagnostic workup in the breast clinic includes mammography, cytology, histology, and immunohistochemistry (IHC; ER, PR, and HER2- receptor testing). All breast carcinomas are histologically confirmed. Treatment available at CHBAH or at other tertiary hospitals in the province is standard breast cancer care, including surgery, chemotherapy, hormonal therapy, and radiation therapy.

The present study includes all women diagnosed with *in situ* or invasive incident breast cancer at CHBAH between 01 October 2006, when routine collection of standardized clinicopathologic data commenced, to 04 July 2012, when data were last extracted. Clinical information and routine histology reports in patient files and electronic pathology reports were used to populate an electronic database that is kept up to date for use in clinical practice. For the present analyses, we obtained an extract of the database, including demographic and clinical variables. Age at diagnosis and date of birth were reported by the patient. Race was recorded by the clinician as “black,” “white,” “colored,” (that is, of mixed race), “Asian,” or “other.” As per the South African Census nomenclature and definitions, these categories refer to peoples with common characteristics in terms of history and descent, especially prior to the 1994 political changes in South Africa [15]. Clinical characteristics included tumor size, lymph-node positivity, stage at diagnosis (primarily coded according to TNM and then converted to Manchester staging), Scarff-Bloom Richardson grade (1, 2, and 3 for well, moderately, and poorly differentiated, respectively), invasiveness, and hormone receptor status (see below). A human immunodeficiency virus (HIV) test was offered to women (enzyme-linked immunosorbent assay (ELISA) method HIV test) at breast cancer diagnosis. Survival and risk-factor data were not routinely collected.

### Immunohistochemistry

The breast clinic’s implemented guidelines include obtaining a core breast biopsy before primary chemotherapy. ER, PR, and HER2 status were routinely measured on these biopsies to inform optimal patient treatment. In practice, >90% of receptor testing was conducted on biopsy material, and the remainder, on mastectomy tissue, but we did not have an indicator of specimen type for individual patients, so we could not use it to perform sensitivity analyses. If a patient underwent preoperative primary chemotherapy, receptor status included here refers to that before such initiation. Tissue biopsies were transferred to an on-site laboratory which is run by the National Health and Laboratory Service (NHLS) of South Africa and is also part of the University Witswatersrand School of Pathology. The NHLS laboratory maintains a close liaison with the breast clinic, and a messenger service ensures rapid delivery of specimens. Time to fixation was <24 hours for biopsy samples and <48 hours for mastectomy tissue. This fully computerized NHLS academic laboratory is accredited by the South African National Accreditation System (SANAS), which performs annual quality-control checks. H&E staining of 3-μm tissue sections was first verified for sufficient numbers of invasive cells and fixation quality. The fully automated immunostainer Ventana Benchmark XT was used for measurement of ER and PR levels (CONFIRM™ , Tucson, Arizona, US).

Staining intensity was scored as 0 (none), 1 (weak), 2 (moderate), or 3 (strong). For ER and PR scores, specimens with <1% nuclei staining were considered negative (score 0), and those with weak (1% to 10%, score 1), moderate (10% to 33%, score 2), and strong (>33%, score 3) staining were classified as hormone-receptor positive, denoted ERP and PRP respectively. HER2 was analyzed by using Ventana (PATHWAY™). No, weak, or moderate HER2 staining (scores 0, 1, 2) were considered HER2 negative (HER2N), and staining intensity 3 as HER2 positive (HER2P), to enable direct comparison with previous studies [3,16,17]. A positive control sample was included in all batches. Breast cancer subtypes were also classified as luminal A (ERP and/or PRP, HER2N), luminal B (ERP and/or PRP, HER2P), HER2-positive enriched (HER2P, ERN, and PRN), and triple-negative (TRN: HER2N, ERN, and PRN), a previously used classification [1,18]. The NHLS laboratory provides the histopathology report to the breast clinic in electronic format; the receptor-staining scores are then entered into the patient database.

### Comparisons with US SEER data

Although the receptor status of breast cancer patients in other settings has no bearing on the management of individual patients in South Africa, international population-level comparisons are informative for the understanding of the wider breast cancer epidemiology. We thus compared age-specific ERN percentages of CHBAH breast cancer patients with those for white and black women in the United States SEER database [19], without making assumptions regarding genetic or other commonalities between the black populations of Soweto and of the United States. We extracted the number of invasive breast cancers diagnosed by ER status (positive, negative, unknown), by 10-year age band, for US white and US black women and for the periods 1992 through 1996 and 2004 through 2008 separately. These two periods correspond to less- and more-intensive mammographic screening, which affects the ERN%. ERN percentages were calculated from those with known ER status (73,022 and 190,695 white and 6,683 and 21,293 black women in the early and later periods, respectively).

Although absolute incidence rates cannot be calculated, distributions of age at diagnosis can be compared between subtypes, as CHBAH patients arise from the same underlying population at risk. Differences in these distributions provide information on the ratios of age-specific incidence rates (for example, dips arise from a slowing of the age-related increases in incidence rates, such as the Clemmesen's hook at the menopause) [20,21].

The study was approved by the University of the Witwatersrand Human Research Ethics Committee (26/08/2011, ref. M110803); the need for individual patient consent was waived, as this was a retrospective record review, which used de-identified data from routine clinical records.

#### ***Statistical analyses***

We first analyzed woman-level and clinical factors associated with the risk of ERN, PRN, and HER2P tumors separately. A generalized linear model for these three binomial responses was fitted, by using a log link function for the linear predictor to obtain regression coefficients that represented the log-risk ratio of the outcome. For analysis of associations with the four-category combined subtypes (luminal A, B, HER2P-enriched, and TRN), we fitted a multivariate logistic regression model to estimate odds ratios of each subtype compared by using the more common luminal A tumors as the reference group. Smoothed distributions of age at diagnosis were plotted by using the Epanechnikov kernel function for density estimation. All models were adjusted for age (<40, 40 to 49, 50 to 59, 60 to 69, 70 to 79, and ≥80 years) and year at diagnosis (2006 to 2007, 2008 to 2009, 2010 to 2012) by using indicator variables for categories. Age was also fitted as a continuous variable (linear trend). These models were first fitted by excluding women with missing receptor status or missing data on other variables on a casewise basis. Given the possibility that “missingness” on receptor status did not occur at random, and may have influenced the overall percentages, the pattern of missingness was examined in relation to age, stage, race, and year at diagnosis by using a logistic regression model and was used to generate 10 imputed values if missing [22]. All analyses were conducted by using STATA version 11.2.

## Results

Over a period of 5 years 9 months, 1,247 breast cancers were diagnosed: 12 (1.0%) in men, and 19 (1.5%) carcinoma *in situ* in women (all were DCIS) were excluded hereafter (Table 1). The present analyses are restricted to the 1,216 women with invasive breast cancer, of whom 90% were black South African women, and the remaining 10% were white, colored, or Asian. Mean age at diagnosis was 55.3 years (standard deviation (SD) 14.3). Of the patients, 23% were diagnosed at stage I/IIA, 24% at stage IIB, and more than half (54%) at stages III/IV. About 89% were moderately/poorly differentiated (grade 2/3) tumors. Clinical notes mentioned ductal carcinoma for 80% of women, lobular in 4.2%, medullary/mucinous in 3.0%, inflammatory in 1.6%, papillary in 2.3%, and Paget disease for 0.6% (data not in tables).

**Table 1:** Characteristics of incident breast cancers diagnosed during October 2006 through July 2012 at Chris Hani Baragwanath Academic Hospital Breast Clinic, Soweto, South Africa

**Characteristic**	**Variable completeness (%)**	**Category**	**No. (column % among nonmissing data)**
Among all incident breast cancers (N = 1247)			
Sex	100	Male	12 (1.0)
		Female	1232 (99.0)
Invasiveness in women	100	Non-invasive	19 (1.5)
		Invasive	1216 (98.5)
Among invasive incident breast cancers in women (N = 1216)			
Ethnicity	99.4	Black	1,092 (90.3)
		White	49 (4.1)
		Colored	46 (3.8)
		Asian	22 (1.8)
Age at diagnosis (years)		<40	182 (15.0)
		40–49	290 (23.9)
		50–59	310 (25.5)
		60–69	221 (18.2)
		70–79	147 (12.1)
		≥80	66 (5.4)
Year of diagnosis	100	2006–2007	172 (14.1)
		2008–2009	431 (35.4)
		2010–2012	613 (50.4)
HIV status	69.0	Negative	686 (81.8)
		Positive	153 (18.2)
Stage	98.1	I	61 (5.1)
		IIA	211 (17.7)
		IIB	280 (23.5)
		IIIA	148 (12.4)
		IIIB-IIIC	385 (32.3)
		IV metastases	107 (9.0)
Tumor grade	80.0	1 = Well-differentiated	106 (10.9)
		2 = Moderately-differentiated	455 (46.8)
		3 = Poorly-differentiated	412 (42.3)
ER	88.2	Negative	376 (35.1)
		Positive: of which	696 (64.9)
		Positive stain score +1	96
		+2	155
		+3	445
PR	87.8	Negative	500 (46.9)
		Positive, of which:	567 (53.1)
		Positive staining score +1	111
		+2	163
		+3	293
HER2	84.9	Negative, of which:	762 (74.1)
		Negative stain score 0	404
		+1	142
		+2	216
		Positive (+3)	267 (26.0)
Subtypes abbreviations:	84.5	Luminal A (ERP and/or PRP, HER2N)	551 (53.7)
		Luminal B (ERP and/or PRP, HER2P)	150 (14.6)
		HER2P enriched (ERN, PRN, HER2P)	117 (11.04)
		Triple negative (ERN, PRN, HER2N)	209 (20.4)

ER, estrogen receptor; PR, progesterone receptor; HER2*,* human epidermal growth factor 2.

### Crude known and unknown receptor status

Among patients with known receptor status, 35% (95% CI, 32 to 38) of tumors were ERN, 47% (44 to 50) PRN, and 26% (23 to 29) HER2P (Table 1). High concordance (82.2%) was found between ER and PR status: percentages jointly classified were ERP and PRP 50.2%, ERP and PRN, 14.9%, ERN and PRP 2.9% and ERN and PRN 32.0%; thus 68% of tumors were hormone receptor positive (ERP and/or PRP). ER, PR, and HER2 status were each missing for between 10% and 15% of women. Missing receptor status and tumor grade tended to occur in the same women (see Additional file 1, Table S1). Receptor status was missing in a nonrandom fashion with respect to other variables (shown for ER status in Additional file 1, Table S1, and is similar but not shown for PR and HER2). Women with later-stage tumors had twice as many missing ER scores than did women with earlier stage I/II tumors. Some suggestion was noted that breast cancers diagnosed at younger than age 40 or older than age 80 had a higher proportion of missing ER status than did patients aged 40 to 80, but the difference was not statistically significant. After imputing missing values, the overall ERN was 35.5% (*n* = 1,192) which was very close to 35.3% in observed data (*n* = 1,063). Similarly, PRN percentages were 47.2% in observed and 47.3 in imputed data: 26.0% for both observed and imputed HER2P. All further results are based on observed data.

### Receptor status, grade, and stage

ER, PR, and HER2 distributions are shown in Table 2, and both crude and adjusted risk ratios for an ERN, PRN, and HER2P tumor associated with other clinical characteristics are provided in Table 3. The greatest ERN and PRN differences occurred with tumor grade: a fourfold greater risk of the tumor being ERN existed if it was grade 3 compared with grade 1 (Table 3). Higher grade was also, but less strongly, associated with an increased risk of HER2P status. Similar to tumor grade, stage III and IV tumors were almost 2 times more likely to be ERN and PRN, but stage was less strongly associated with HER2 status (Table 2). Consequently, both higher grade and later stage were strongly associated with the combined subtype. HER2P-enriched (66%) and 58% of TRNs were diagnosed at stages III/IV compared with 47% of luminal A tumors. Both HER2P-enriched and TRN subtypes had >60% grade 3 tumors, compared with 28% of luminal A.

**Table 2:** Distribution of ER, PR, HER2, and their combinations by woman and tumor characteristics

		**ER status**			**PR status**			**HER2 status**			**ER, PR, and HER2 status number of women**				**ER, PR, and HER2 status row distribution (%)**			
		**Number positive**	**Number negative**	**% ER negative**	**Number positive**	**Number negative**	**% PR negative**	**Number positive**	**Number negative**	**% HER2 positive**	**Luminal A**	**Luminal B**	**HER2P-enriched**	**TRN**	**Luminal A**	**Luminal B**	**HER2P-enriched**	**TRN**
All women		688	375	35.3†	559	499	47.2†	756	265	26.0†	545	149	116	209	53.5	14.6	11.4	20.5
Age	<40	100	54	35.1	84	71	45.8	102	48	32.0	77	29	19	25	51.3	19.3	12.7	16.7
	40–49	177	85	32.4	148	113	43.3	173	77	30.8	128	47	30	45	51.2	18.8	12.0	18.0
	50–59	149	121	44.8	107	162	60.2	199	59	22.9	123	28	31	75	47.9	10.9	12.1	29.2
	60–69	134	58	30.2	106	83	43.9	141	42	23.0	109	22	20	31	59.9	12.1	11.0	17.0
	70–79	90	41	31.3	82	49	37.4	96	31	24.4	74	19	12	22	58.3	15.0	9.5	17.3
	≥80	38	16	29.6	32	21	39.6	45	8	15.1	34	4	4	11	64.2	7.6	7.6	20.8
Calendar year	2006–2007	86	60	41.1	72	72	50.0	87	37	29.8	66	16	21	21	53.2	12.9	16.9	16.9
	2008–2009	241	144	37.4	186	198	51.6	263	116	30.6	183	65	51	80	48.3	17.2	13.5	21.1
	2010–2012	361	171	32.1	301	229	43.2	406	112	21.6	296	68	44	108	57.4	13.2	8.5	20.9
Stage	I	43	13	23.2	41	16	28.1	40	14	25.9	34	10	4	6	63.0	18.5	7.4	11.1
	II	319	130	29.0	262	184	41.3	345	95	21.6	262	60	35	81	59.8	13.7	8.0	18.5
	III	271	202	42.7	218	254	53.8	314	133	29.8	207	68	65	107	46.3	15.2	14.5	23.9
	IV	55	30	35.3	38	45	54.2	57	23	28.8	42	11	12	15	52.5	13.8	15.0	18.8
Tumor grade	1	89	12	11.9	76	25	24.8	84	15	15.2	76	12	3	8	76.7	12.1	3.0	8.1
	2	331	109	24.8	278	161	36.7	332	96	22.4	276	60	36	55	64.6	14.1	8.4	12.9
	3	189	207	52.3	148	246	62.4	264	125	32.1	138	61	64	125	35.6	15.7	16.5	32.2
	Missing	79	47	37.3	57	67	54.0	76	29	27.6	55	16	13	21	52.4	15.2	12.4	20.0
Ethnicity	Black	606	351	36.7	492	461	48.4	679	240	23.1	481	131	109	196	52.5	14.3	11.9	20.4
	White	32	8	20.0	27	13	32.5	29	9	23.7	24	7	2	5	63.2	18.4	5.3	13.2
	Colored	30	9	23.1	26	13	33.3	30	8	21.1	26	5	3	4	68.4	13.2	7.9	10.5
	Asian	15	5	36.7	12	8	40.0	14	5	25.3	11	4	1	3	57.9	21.1	6.3	15.8

Note: Percentages are among women with known receptor status. Numbers missing are provided in Table 1.

†Percentages (95% confidences intervals) are 35.3 (32.4 to 38.2) for ERN, 47.2 (44.2 to 50.2) for PRN, and 26.0 (23.3 to 28.6) for HER2P.

**Table 3:** Risk ratio for ERN, PRN, and HER2P breast cancer

		**Risk ratio of ERN breast cancer**			**Risk ratio of PRN breast cancer**			**Risk ratio of HER2P breast cancer**		
**Factor**	**Category**	**Crude RR**	**Adjusted RR**		**Crude RR**	**Adjusted RR**		**Crude**	**Adjusted RR**	
			**RR**	**95% CI**		**RR**	**95% CI**		**RR**	**95% CI**
Age (years)	<40	1.08	1.11	0.84, 1.45	1.06	1.06	0.85, 1.32	1.04	1.02	0.76, 1.38
	40–49 (ref)	1	1	-	1	1	-	1	1	-
	50–59	1.38	1.33	1.08, 1.65	1.39	1.37	1.16, 1.61	0.74	0.74	0.56, 0.99
	60–69	0.93	0.92	0.70, 1.20	1.01	1.03	0.83, 1.26	0.75	0.75	0.54, 1.03
	70–79	0.96	0.95	0.70, 1.29	0.86	0.87	0.67, 1.13	0.79	0.80	0.56, 1.15
	≥80	0.91	0.89	0.57, 1.38	0.92	0.86	0.60, 1.23	0.49	0.49	0.25, 0.94
	per 5 years	*0.99*	0.99	*0.95, 1.02*	0.99	0.99	*0.97, 1.01*	0.95	0.95	*0.92, 0.99*
				*P*_*t*_*= 0.37*			*P*_*t*_*= 0.27*			*p*_*t*_*= 0.01*
Calendar year	2006–2007	1.10	*1.05*	*0.84, 1.32*	0.97	0.91	*0.76, 1.08*	0.97	0.94	0.69, 1.28
	2008–2009 (ref)	1	*1*	*-*	1	1	*-*	1	1	-
	2010–2012	0.86	*0.84*	*0.71, 1.00*	0.84	0.82	*0.72, 0.94*	0.71	0.70	0.56, 0.88
				*P = 0.03*			*P*_*t*_*= 0.09*			*p = 0.01*
Stage	I (ref)	1	1	-	1	1	-	1	1	-
	II	1.25	1.23	0.75, 2.02	1.47	1.55	1.00, 2.41	0.83	0.89	0.54, 1.44
	III	1.84	1.76	1.08, 2.85	1.92	1.99	1.29, 3.09	1.15	1.22	0.76, 1.98
	IV	1.52	1.48	0.85, 2.57	1.93	1.92	1.20, 3.07	1.11	1.19	0.67, 2.11
				*P*_*t*_*= 0.001*			*P*_*t*_*= 0.001*			*p*_*t*_*= 0.05*
Tumor grade	1 (ref)	1	1	-	1	1		1	1	-
	2	2.09	1.94	1.11, 3.37	1.48	1.42	0.99, 2.04	1.48	1.30	0.79, 2.15
	3	4.40	3.95	2.30, 6.79	2.52	2.37	1.67, 3.36	2.12	1.80	1.10, 2.95
	Missing	3.14	2.83	1.58, 5.04	2.18	1.96	1.34, 2.86	1.82	1.63	0.93, 2.86
										*p = 0.01*
Ethnicity	Black (ref)	1	1	-	1	1	-	1	1	-
	Nonblack^a^	0.61	0.61	0.42, 0.89	0.71	0.73	0.55, 0.95	0.89	0.86	0.59, 1.25
				*P = 0.009*			*P = 0.02*			*p = 0.42*
	White	0.55	0.55	0.29, 1.01	*0.67*	0.69	0.44, 1.07	0.91	0.87	0.49, 1.55
	Colored	0.63	0.63	0.35, 1.11	*0.69*	0.70	0.45, 1.09	0.81	0.77	0.41, 1.43
	Asian	0.68	0.72	0.34,1.53	*0.83*	0.81	0.48, 1.38	1.01	0.99	0.47, 2.09
				*P = 0.08*			*P = 0.13*			*p = 0.86*

Note: Analyses based on 1,063 women for ER, 1,058 for PR, and 1,021 for HER2. Adjusted RRs are adjusted for age, stage, year, and race, unless this is the variable of interest. *P* values denote test of heterogeneity across categories; *P*_t_ denotes test of linear trend.

^a^Nonblack women are the combined group of white, colored, and Asian women.

### Time trends

**Figure 1:**
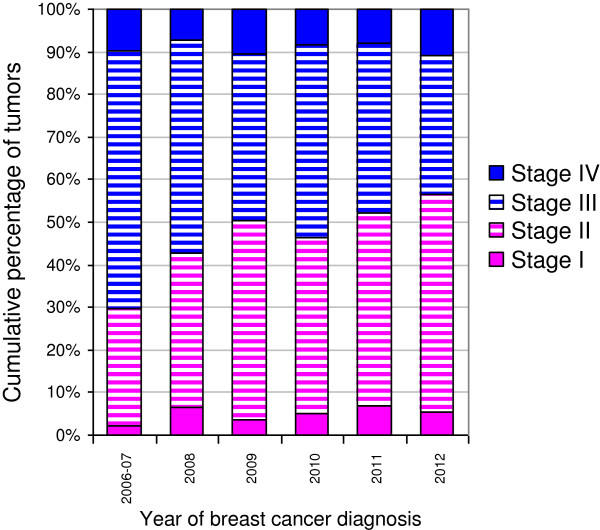
Relative distribution of stage at diagnosis by year at diagnosis.

A time-trend of a declining proportion of ERN tumors was accounted for by the trend of earlier stage at presentation over time. From 2006–2007 to 2010–2012, the percentage of tumors diagnosed at stages III/IV declined from 66% to 46% (Figure 1) and the percentage of poorly differentiated tumors decreased from 55% to 35%. The trend of earlier presentation did not differ by age, race, or subtype (data not shown).

### Race

After adjusting for age, year, and stage, nonblack women had a 39% lower risk of having an ERN tumor, a 29% lower risk of a PRN tumor compared with black patients, but no significant difference in HER2 status (Table 3). Compared with 11% of tumors that were HER2P enriched and 20% TRNs in black breast cancer patients, corresponding values were 5% and 13% in white, 8% and 10% in colored, and 5% and 16% in Asian women (Table 2). After adjusting for age and stage, nonblack women had approximately half the odds of a HER2 tumor compared with having a luminal A tumor (OR, 0.40 (0.17 to 0.96) and half the odds of a TRN tumor (OR, 0.47, 0.24 to 0.89) than did black women (Table 4). These differences were also present before adjustment for age and stage, because nonblack and black women did not differ in age at diagnosis (mean ages, 54.1 years (SD, 13.3) and 55.3 (SD, 14.6) respectively, *P* = 0.36) and stage at diagnosis (percentages diagnosed at stages I/II, III, and IV being 47.0, 42.7, and 10.3 in nonblack and 46.4, 45.1, and 8.5 in black women; *P* = 0.74).

**Table 4:** **Adjusted odds ratios for Luminal B, HER2**^**+ **^**enriched, and triple-negative tumors compared with having a Luminal A tumor**

		**Luminal B (*n *** **= 148) vs Luminal A (*n *** **= 545)**	**HER2P enriched (*n *** **= 116) vs Luminal A (*n *** **= 545)**	**Triple negative (*n *** **= 209) vs Luminal A (*n *** **= 545)**
		**Odds ratio (95% CI)**	**Odds ratio (95% CI)**	**Odds ratio (95% CI)**
Age (years)	<40	1.04 (0.60, 1.79)	1.11 (0.58, 2.13)	0.94 (0.53, 1.65)
	40–49	1	1	1
	50–59	0.61 (0.36, 1.04)	1.08 (0.61, 1.91)	1.70 (1.09, 2.67)
	60–69	0.54 (0.31, 0.96)	0.75 (0.40, 1.41)	0.78 (0.46, 1.33)
	70–79	0.69 (0.38, 1.28)	0.69 (0.33, 1.46)	0.84 (0.47, 1.52)
	≥80	0.30 (0.10, 0.91)	0.50 (0.16, 1.53)	0.84 (0.39, 1.81)
		*P*_*trend*_*= 0.003*	*P*_*trend*_*= 0.21*	*P*_*trend*_*= 0.33*
Calendar year	2006–2007	1	1	1
	2008–2009	1.57 (0.83, 2.91)	0.93 (0.51, 1.69)	1.35 (0.76, 2.39)
	2010–2012	1.00 (0.54, 1.85)	0.49 (0.27, 0.89)	1.12 (0.64, 1.94)
		*P*_*trend*_*= 0.27*	*P*_*trend*_*= 0.003*	*P*_*trend*_*= 0.96*
Stage	I–II	1	1	1
	III–IV	1.41 (0.98, 2.04)	2.34 (1.53, 3.59)	1.67 (1.20, 2.32)
Tumor grade	1	1	1	1
	2	1.16 (0.59, 2.31)	2.54 (0.75, 8.59)	1.62 (0.73, 3.60)
	3	2.35 (1.17, 4.73)	8.65 (2.59, 28.9)	7.70 (3.52, 16.8)
	Missing	1.72 (0.74, 4.01)	4.61 (1.23, 17.26)	2.99 (1.21, 7.37)
Ethnicity	Black	1	1	1
	Nonblack^a^	0.95 (0.52, 1.71)	0.40 (0.17, 0.96)	0.47 (0.24, 0.89)
	White	1.02 (0.42, 2.44)	0.34 (0.08, 1.49)	0.50 (0.19, 1.35)
	Colored	0.70 (0.26, 1.91)	0.44 (0.13, 1.53)	0.36 (0.12, 1.07)
	Asian	1.37 (0.41, 4.30)	0.41 (0.05, 3.27)	0.63 (0.17, 2.32)

Note: Odds ratios are adjusted for age, stage, year, and race, unless this is the variable of interest.

^a^Nonblack women are the combined group of white, colored, and Asian women.

**Figure 2:**
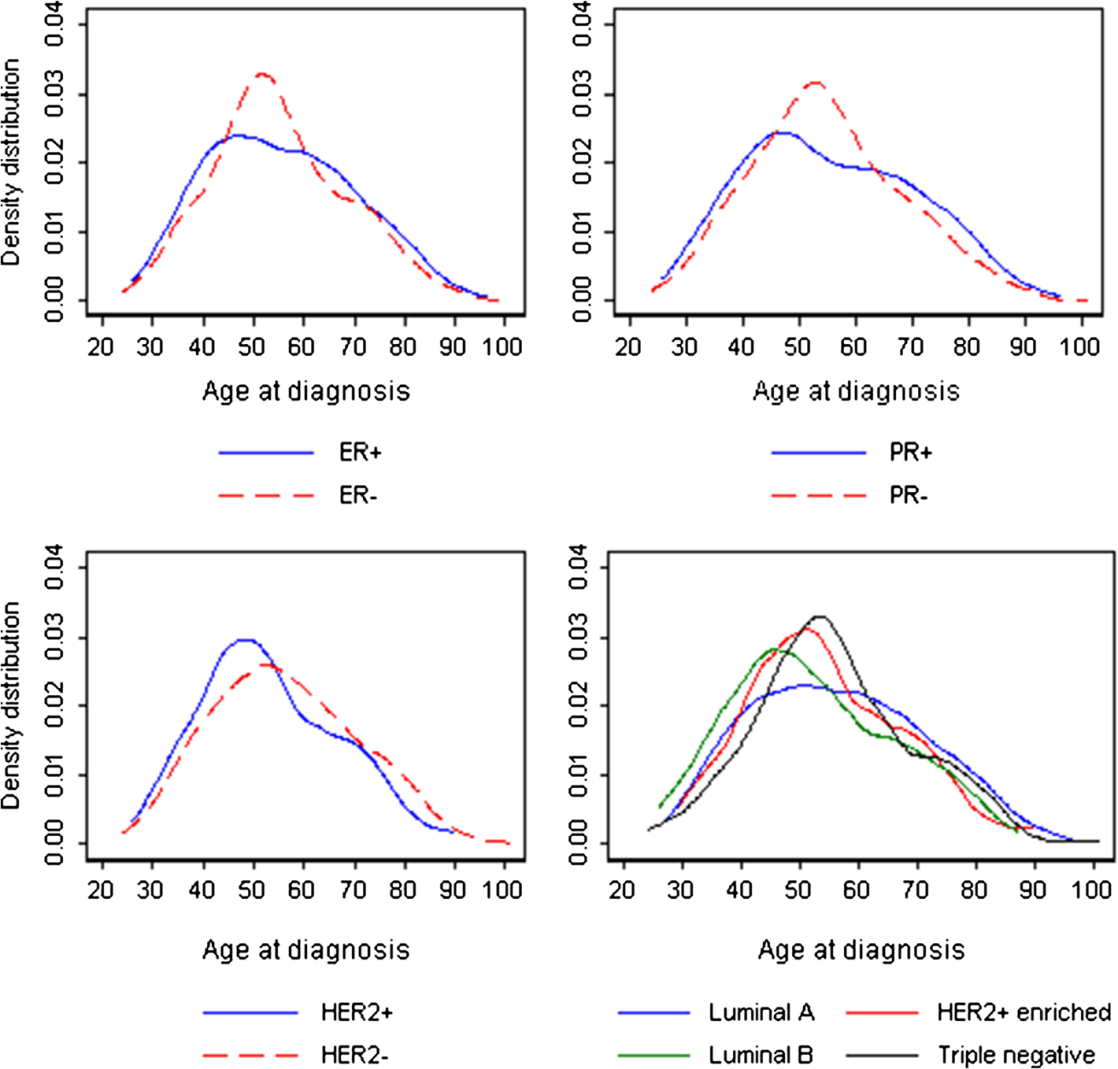
Smoothed frequency distributions of age at breast cancer diagnosis in black South African women, by receptor status.

**Figure 3:**
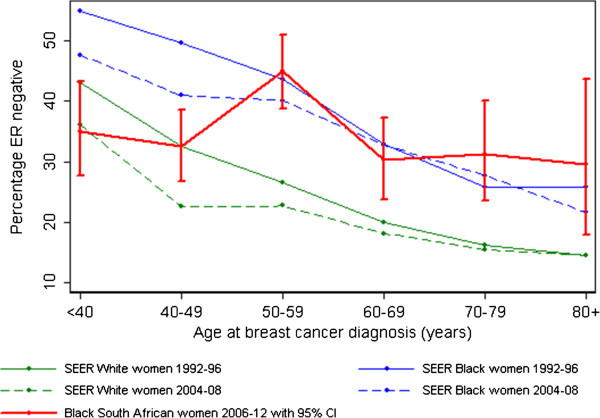
Age-specific percentages of breast tumors that are ER negative in black women in Soweto (2006–2012) and US black and white breast cancer cases diagnosed during 1992–1996 and 2004–2008.

Owing to these suggestions of racial variations in ER status, subsequent Figures 2 and 3 were restricted to black women only (that is, the more homogeneous group and population of main interest).

### Age

Age-at-diagnosis distributions by subtype are shown in Figure 2 among black patients. ERP, PRP, and HER2P tumors all had peak incidences in the middle to late forties and a slight dip in the mid-fifties, indicating deceleration of the rate of increase of the underlying incidence rates. A dome-shaped distribution was found for luminal A tumors, and peaks followed by troughs for luminal B, HER2P enriched, and TRNs. Median age at diagnosis was youngest for luminal B tumors (49.6 years that is, >5 years younger than for luminal A tumors (55.0 years)). HER2P tumors were also diagnosed at younger ages than were TRN tumors. These arise from the trend of older age associated with a lower risk of being HER2P (Table 3), whereas age was not strongly associated with ER or PR status.

### Comparison with US SEER data

Figure 3 plots age-specific ERN percentages in CHBAH black patients overlaid on those for US SEER black and white women in an early (1992 to 1996) and recent (2004 to 2008) period. The lack of a strong inverse trend of ER negativity with age in the CHBAH data contrasts with the age-related decline that has been observed in the US in earlier and later periods. ERN percentages in South Africa were more similar to those of white women younger than 50 years, but at ages 50+, they coincide with those of US black women (age-adjusted odds ratio for ER negativity comparing black South African with US black women older than age 50 were 1.05 (95% CI, 0.89 to 1.26) in the early and 1.12 (0.95 to 1.33) in later periods.

The high proportion of HIV^+^ (17%) breast cancer patients did not influence any of the results presented (an HIV focus is the subject of a separate article). Most HIV^+^ women had a simultaneous diagnosis of HIV and breast cancer; thus they had not been taking antiretroviral medication prior to breast cancer diagnosis.

## Discussion

In this large systematic study of breast cancer receptor status measured at diagnosis in a South African public hospital, we observed that (a) the majority (63%) of tumors were ER positive in black breast cancer patients, and triple-negatives constituted one fifth of tumors, that is, an overall subtype distribution not excessively different from that in the West, especially to that of US black women older than 50; (b) black women were more likely than nonblack women to have ERN or triple-negative breast cancer, and PRN and HER2P proportions were relatively high; (c) in the absence of any organized screening program, a decline occurred in the proportion of late-stage breast cancers in a population that previously had a very high proportion of late-stage presentation, suggesting possibilities for downstaging in low-resource settings. These combined observations indicate that very late-stage aggressive tumors may not be inherent features of the breast cancer burden throughout SSA. They suggest that, combined with appropriate and timely treatment, improvements in breast cancer survival rates may be realistic targets in this and comparable settings.

Based on >1,000 receptor-characterized tumors, ER positivity (65% overall and 63% in black women) in South African breast cancer patients was consistent with that of black American women older than 50 years and very similar to a Nigerian study (65%), which was also based on IHC at diagnosis, and to others in South Africa and Sudan [3-5]. However, several African studies have reported that fewer than 40% of tumors were ERP [6-9,23]. One of the latter studies also observed that TRNs constituted >50% of tumors, compared with 20% observed here.

Several factors may contribute to these differences, including the age of breast cancer patients, stage at diagnosis, histopathologic methods, differential underlying risk-factor distributions, and ERP and ERN incidence rates and genetic heterogeneity across this vast continent. The average age at breast cancer diagnosis of 55 years in the present study is between 6 and 10 years older than that in most previous studies of receptor status from SSA [3,6-9] and may contribute to the lower ERN proportion in our study, although ERP tumors dominated even at premenopausal ages in Soweto. As expected, more late-stage tumors were ERN, as such tumors have more-aggressive growth, and tumor progression is associated with a loss of estrogen expression [24]. The latter factor may have also contributed to the lower ERN proportion in our study than in other SSA studies, as stage at diagnosis was earlier (54% stage III/IV in Soweto versus >80% in other studies). As the study design in a case series and incidence rates cannot be calculated, the lower ERN proportion in Soweto than in some other SSA settings may result from similar incidence rates of ERN breast cancer, but a higher incidence rate of ERP disease in Soweto. Urban South African women are more westernized than their counterparts elsewhere in Africa (for example, smaller family size, greater use of hormonal contraception) [25], which may have led to real increases in incidence rates for ERP breast cancer and thus to a larger total breast cancer burden with a greater proportion of ERP cases. Differences in findings within Africa may also have been influenced by specimen collection or storage conditions (detailed in the introduction). Notably, the studies with IHC conducted at diagnosis that are less affected by antigen degradation have shown that ERP tumors predominate. Additionally, nuclear-staining cutoffs in some historical studies were 10% or 11% [3,8], as per the guidelines at the time, whereas 1% was used here. Applying the higher cutoff to our data, the ERN percentage would have increased from 35% to 43%. Determination of receptor-status relative frequencies in other African settings, including rural populations, is needed. Furthermore, as no single outright majority subtype exists, receptor classification is needed for clinical decision making, but is not always available in SSA. That the majority of tumors in this study were the better-prognosis ERP tumors is, meanwhile, an encouraging feature of this burden. Research is needed into whether the more-favorable prognostic profile in Sowetan breast cancer patients confers survival gains.

Although the majority breast cancer burden in this study was postmenopausal and ERP, nevertheless, one in three tumors was poorer-prognosis HER2P enriched or TRNs. The real HER2P percentage may be even higher, as 26% of tumors had a 2^+^ IHC HER2 staining score, and previous unpublished work at CHBAH comparing IHC and FISH HER2 status found that 50% of IHC HER2 2+ stains were FISH HER2P. Regardless of the HER2 assessment method, the percentage of HER2P tumors is higher than in several other reports in Africa [3,7]. Thus, affordable treatment for HER2P tumors, by trastuzumab or other agents, is needed in this setting. Additionally, the proportion of tumors that were PRN (48%) was higher than in US data today and before the introduction of screening (33% to 39% PRN at age 50) [26]. Reexamination of these proportions in Africa, and an investigation of their drivers, are needed.

A predominance of bettERNprognosis luminal A and B tumors and the strong downstaging trend over time suggest that late stage at diagnosis in the South African setting may be driven to a greater extent by nonbiologic determinants of stage at diagnosis rather than by the predominance of an inherent aggressive rapidly growing tumor, but must be confirmed in other studies. Luminal A and B tumors, both ERP tumors, have good 5-year survival (about 90%) compared with <80% for HER2P and TRN tumors in US settings [18]. Whether subtype-specific tumors have different prognoses still must be investigated within African populations, but given the downstaging trends observed in this South African setting, the potential impact on lives saved can be estimated, assuming external stage-specific 5-year survival rates [27]. Of 1,000 women diagnosed with this improvement would result in an additional 80 women alive 5 years after diagnosis. The reasons for the observed downstaging are likely to be multifactorial, including factors at both the individual and health-system levels (for example, improving public awareness through media campaigns, faster referral, and a dedicated tertiary hospital breast clinic that was increasing in volume and ease of access). Importantly, these trends occurred within a resource-limited setting and without population-based screening, and demonstrate that earlier presentation is achievable in similar settings.

Further research is needed to evaluate the relative contributions to downstaging of all components of a woman’s journey from first noticing symptoms to diagnosis at CHBAH.

Black women were more likely to have ERNnegative tumors than were white, colored, or Asian South African breast cancer patients. Despite wide confidence intervals, the odds ratios for TRN, HER2P enriched, and luminal B versus luminal A tumors for black versus nonblack women were 2.0 (95% CI, 1.1 to 3.8), 2.2 (1.0 to 4.9), and 1.0 (0.6 to 1.8) (reciprocals of those already provided in Table 4), estimates that are remarkably similar to those found for African American versus non-African American women in the Carolina Breast Cancer Study (ORs of 2.1, 1.8, and 0.9, respectively) [18]. Racial differences in the South African study are unlikely to be due to differential early detection by screening mammography because women with medical aid coverage of screening mammograms are unlikely to be directed to CHBAH for diagnosis. Women with positive findings on screening mammography are likely to remain within the private sector for diagnostic workup and treatment. Indeed, all the women for whom we had referral information had been referred from a local health clinic or doctor, hospital, or were self-referrals.

The age distributions for ERN and PRP tumors in Soweto showed a dip in frequency distribution at age 60, which corresponds to the Clemmesen hook and reflects a plateauing of incidence rates at postmenopausal ages [20,21]. HER2P tumors also displayed this feature of a younger age peak at age 50 and a dip at age 60; their age distribution was virtually equivalent to quite a distinct population (for example, in Hawaii [17]). However, differences in age distributions by ER-receptor status were not as pronounced as have been observed in high-risk populations such as the United States (younger distribution for ERN tumors) [17,21]; thus ER proportions by age would benefit from re-investigation in other large breast cancer case series from Africa. Higher rates of ERP cancers at older ages in the United States are likely to account for this difference, because the major lifestyle transitions (early menarche, low parity, late childbearing, postmenopausal weight gain, less breastfeeding) are stronger risk factors for ERP breast cancer, and screening is more likely to detect these tumors [28]. In US, white and black women incidence rates of ERP breast cancer have increased, whereas for ERN tumors, they have decreased during the past 2 decades [10], driving an increasing percentage of ERP cancers over time and with age. In the same way, South Africa may be at an earlier stage of a breast cancer transition that will see an increasing rate of ERP disease as younger cohorts with less-traditional reproductive profiles reach postmenopausal ages.

This study is unique for this setting in terms of the sample size, IHC performed at diagnosis on prechemotherapy biospecimens in quality-controlled laboratories, and inclusion of HER2 expression. Analytically, we carefully assessed the influence of the 11% unknown receptor status; they were more likely to be ERN, as they were advanced tumors. The study is essentially a hospital-based case series, but it is likely to have fewer problems of underdiagnosis and thus a skewed patient profile that misrepresents the true population burden in rural and in lower-resource settings. The case series is likely to be representative of breast cancers in the public health sector of Soweto, as CHBAH provides affordable care and is easily accessible and known to the geographically close population (78% live within 25 km).

## Conclusion

Although a greater proportion of black than nonblack South African women had ERN or TRN breast cancer, in all racial groups in this urban South African setting, breast tumors were predominantly ERP. We observed a strong trend of earlier stage at diagnosis over a 5–year period. Further research is needed to assess subtype-specific risk factors and subtype-specific survival in this setting. These findings provide initial indications that late-stage aggressive breast cancers may not be an inherent feature of the sub-Saharan African breast cancer burden.

## Abbreviations

CHBAH: Chris Hani Baragwanath Academic Hospital; CI: Confidence interval; ER: Estrogen receptor; HER2: Human epidermal growth factor 2; IHC: Immunohistochemistry; OR: Odds ratio; PR: Progesterone receptor; RR: Risk ratio; SEER: Surveillance Epidemiology and End Results program; TRN: Triple negative.

## Competing interests

The authors declare that they have no conflicts of interests.

## Authors’ contributions

HC and MJ initiated and managed data collection for the study, clinical data were collected by HC and NB. EvdB ensured and provided immunohistochemistry results. JJ, AN, IdSS, IR, and JS contributed to the interpretation of the results. VM had the study concept, analyzed the data, and wrote the manuscript. All authors read and approved the final manuscript.

## Supplementary Material

Additional file 1: Table S1 Distribution of unknown ER status across clinical characteristics. Click here for file
