# Supplementary material for: Breast cancer receptor status and stage at diagnosis in over 1,200 consecutive public hospital patients in Soweto, South Africa: a case series
Source: Breast Cancer Res. 2013 Sep 17;15(5):R84. doi: 10.1186/bcr3478 (PMC3978918; doi:10.1186/bcr3478)
Supplement: Additional file 1: Table S1 — Distribution of unknown ER status across clinical characteristics. [file bcr3478-S1.doc]

**Supplementary Table 1. Percentage of hormone status unknown according to woman and clinical factors**

| **Factor** | **Category** | **Number of women** | **Unknown ER status**  **N (%)** | | **p-value**^c^ |
| --- | --- | --- | --- | --- | --- |
|  |  |  | N | % |  |
|  | All patients | 1192 |  |  |  |
| Age | <40  40-49  50-59  60-69  70-79  80+ | 177  287  304  217  142  65 | 23  25  34  25  11  11 | 13.0  8.7  11.2  11.5  7.8  16.9 | *p =0.28* |
| Calendar year | 2006-07  2008-09  2010-12 | 171  428  593 | 25  43  61 | 14.6  10.1  10.3 | *p=0.22* |
| Stage | I  II  III  IV | 61  491  533  107 | 5  42  60  22 | 8.2  8.6  11.3  22.6 | *p=0.003* |
| Tumor grade | 1  2  3  Missing | 104  451  410  227 | 3  11  14  101 | 2.9  2.4  3.4  44.5 |  |
| Ethnicity | Black  Non-Black^d^  NK  Non-black: White  Coloured  Asian | 1071  114  7  47  45  22 | 114  15  0  7  6  2 | 10.6  13.2  0  14.9  13.3  9.1 | *p=0.76* |

^c^ p-values denote test of heterogeneity across categories; p_trend_ denotes test of linear trend.

^d^ White, Coloured and Asian women.
